# The Antimicrobial Peptide C14R Is Active Against All Pathogenic Species of the ESKAPE Group

**DOI:** 10.3390/antibiotics15020211

**Published:** 2026-02-15

**Authors:** Daniel Gruber, Verena Vogel, Jan-Christoph Walter, Grigory Bolotnikov, Armando Rodríguez, Nico Preising, Ludger Ständker, Carolina Firacative, Barbara Spellerberg, Ann-Kathrin Kissmann, Frank Rosenau

**Affiliations:** 1 Institute of Pharmaceutical Biotechnology, Ulm University, 89081 Ulm, Germany; daniel.gruber@uni-ulm.de (D.G.); jan-christoph.walter@uni-ulm.de (J.-C.W.); grigory.bolotnikov@uni-ulm.de (G.B.); 2 Institute of Medical Microbiology and Hygiene, University Clinic of Ulm, Frauensteige 12, 89075 Ulm, Germany; verena-1.vogel@uni-ulm.de (V.V.); barbara.spellerberg@uniklinik-ulm.de (B.S.); 3 ULMTeC Core Facility for Functional Peptidomics, Faculty of Medicine, Ulm University, 89081 Ulm, Germany; armando.rodriguez-alfonso@uni-ulm.de (A.R.); nico.preising@uni-ulm.de (N.P.); ludger.staendker@uni-ulm.de (L.S.); 4 ULMTeC Core Facility of Mass Spectrometry and Proteomics, Faculty of Medicine, Ulm University, 89081 Ulm, Germany; 5 Studies in Translational Microbiology and Emerging Diseases (MICROS) Research Group, School of Medicine and Health Sciences, Universidad de Rosario, Bogota 111221, Colombia; cfiracative@gmail.com; 6 Laboratory for Life Sciences and Technology (LiST), Faculty of Medicine and Dentistry, Danube Private University, 3500 Krems an der Donau, Austria

**Keywords:** ESKAPE pathogens, antimicrobial activity, C14R peptide

## Abstract

The global rise in antimicrobial resistance among the ESKAPE pathogens represents a major challenge to public health. Here, we report the broad-spectrum antibacterial activity of the synthetic antimicrobial and pore-forming peptide C14R against all six ESKAPE species. Using a radial diffusion assay and resazurin-based viability testing, C14R exhibited a potent bactericidal effect with minimum inhibitory concentrations (MICs), defined as the lowest concentration of an antimicrobial agent that completely inhibits visible growth of planktonic microorganisms, ranging from 3.4 µg/mL (*Enterococcus faecium*, vancomycin-resistant) to 45.2 µg/mL (*Klebsiella quasipneumoniae*, ESBL). C14R also inhibited biofilm formation by Gram-positive pathogens, with minimum biofilm inhibitory concentrations (MBICs), referring to the minimal concentration required to prevent the development of biofilms, of 15.0 µg/mL (*Staphylococcus aureus*, MRSA) and 22.0 µg/mL (*E. faecium*, VRE), whereas Gram-negative biofilms showed higher tolerance. Together, these findings demonstrate that C14R retains high activity against multidrug-resistant ESKAPE strains, highlighting its potential as a lead compound for the development of next-generation antimicrobial drugs to expand the portfolio of available antibiotics and brace health systems against emerging severe infections.

## 1. Introduction

The Infectious Diseases Society of America (IDSA) reported “Bad Bugs, No Drugs” and its “Call to Action”, which highlighted the threat posed by ESKAPE pathogens (*Enterococcus faecium*, *Staphylococcus aureus*, *Klebsiella quasipneumoniae*, *Acinetobacter baumannii*, *Pseudomonas aeruginosa*, and *Enterobacter*-species). While their foresight deserves recognition, the persistence of these organisms as a major problem today underscores the urgent need to accelerate the discovery and development of new antibacterial drugs [1,2,3]. Multidrug-resistant (MDR) bacteria, especially the ESKAPE pathogens, are a critical global challenge, causing difficult-to-treat nosocomial infections [4,5]. They require urgent new solutions for infection control, as they often “escape” the effect of commonly used antimicrobial drugs [6,7]. In fact, the World Health Organization (WHO) listed all six of the ESKAPE pathogens in its “2024 Bacterial Priority Pathogen List (WHO BPPL)”, underscoring the relevance of finding treatment alternatives, whereas rising antimicrobial resistance is not only a problem for third-world countries but also challenges healthcare in first-world countries [8,9]. The process of “escaping” the effects of the antimicrobial agents relies on multiple resistance mechanisms the microorganisms developed over time. Resistance against vancomycin is frequently observed, as seen in vancomycin-resistant *E. faecalis* (VRE). Vancomycin binds to a precursor involved in peptidoglycan maturation, preventing penicillin-binding proteins (PBPs) from cross-linking lipid II into mature peptidoglycan, thereby weakening the structural integrity of the cell envelope [10,11,12]. In methicillin-resistant *S. aureus* (MRSA), methicillin resistance is mediated by the *mecA* gene, which encodes penicillin-binding protein 2A (PBP2A), characterized by its reduced affinity for β-lactam antibiotics [13,14,15]. MRSA can compensate the inhibited PBP1 activity by utilizing the “novel” PBP2A, thereby maintaining peptidoglycan cross-linking and enabling growth even in the presence of β-lactam antibiotics such as methicillin [16,17]. *K. pneumoniae* and *Escherichia coli* are frequently found to carry extended-spectrum β-lactamases (ESBLs), making both clinically relevant strains [18,19,20]. These enzymes hydrolyze the β-lactam ring of a wide range of β-lactam antibiotics, including the so-called “third-generation cephalosporins” [21,22,23]. In the case of *Acinetobacter* spp. MDR is enhanced by horizontal gene transfer of plasmids, transposons and integrons carrying multiple antibiotic genes, as well as by low outer membrane permeability and efflux pumps that reduce drug accumulation [24,25,26]. *P. aeruginosa* exhibits intrinsic resistance to many antibiotics through low outer membrane porin permeability, active efflux pump systems and chromosomally encoded AmpC β-lactamase, with resistance further enhanced by mutations or acquisition of carbapenemase genes, ultimately leading to MDR strains [2,27,28,29]. Antimicrobial peptides (AMPs) represent an evolutionarily conserved component of innate immunity and are increasingly investigated as alternatives to conventional antibiotics [30,31]. Typically short, cationic and amphipathic, AMPs interact preferentially with negatively charged microbial membranes, leading to membrane disruption, pore formation or intracellular target interference [32,33]. Due to their rapid and multitarget mode of action, AMPs are considered less prone to resistance development compared to classical antibiotics [34,35]. The synthetic antimicrobial peptide C14R is a rationally designed, pore-forming AMP that has been extensively characterized in previous studies. C14R is a short cationic and amphipathic 2 kDa peptide consisting of 16 amino acid residues [36]. It exhibits strong antibacterial activity against *Pseudomonas aeruginosa* and low cytotoxicity toward mammalian cells [36]. The C14R peptide also has been demonstrated to show broad antifungal activity against multiple *Candida* and *Cryptococcus* species. Furthermore, antibiofilm effects of C14R against selected fungal pathogens have been reported [37,38,39]. Although the antimicrobial activity and pore-forming mechanism of C14R have been demonstrated previously against selected bacterial and fungal pathogens, a systematic evaluation of its efficacy against the complete ESKAPE pathogen group under standardized experimental conditions has been lacking. In this study a comprehensive, side-by-side analysis of the antibacterial and antibiofilm activity of C14R against all six clinically relevant ESKAPE species, including multidrug-resistant reference strains, using harmonized and directly comparable assay conditions is shown. This systematic approach defines both the activity spectrum and the limitations of C14R in the context of high-priority nosocomial pathogens and provides a clinically relevant reference framework for evaluating antimicrobial peptides as potential alternatives or complements to conventional antibiotics in the fight against multidrug-resistant infections. C14R was selected for evaluation against ESKAPE pathogens due to its previously demonstrated membrane-targeting mechanism, low cytotoxicity toward mammalian cells and broad activity across bacteria and also fungal species, making it a promising candidate for addressing multidrug-resistant nosocomial infections.

## 2. Results

**Figure 1:**
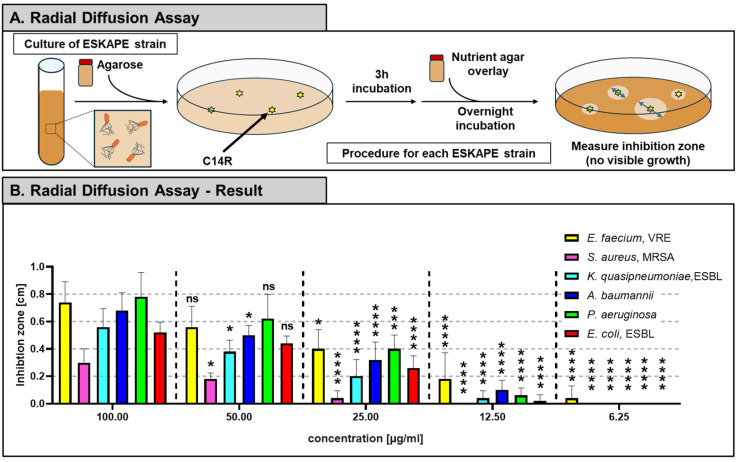
Determination of the antimicrobial activity of C14R using a two-layer radial diffusion assay. (**A**) Schematic illustration of the two-layer radial diffusion assay. Target bacterial strains were embedded in a first agarose layer and wells were punched into the solidified medium. Wells were filled with serial dilutions of the antimicrobial peptide C14R (100, 50, 25, 12.5 and 6.25 µg/mL). After 3 h of pre-incubation at 37 °C, a second layer of Trypticase soy agar was poured on top. Plates were incubated overnight at 37 °C. Blue double-headed arrows indicate the diameter of the inhibition zones, which was measured for the subsequent results. (**B**) Measurement of the inhibition zones (cm) for ESKAPE strains at different C14R concentrations. Data are represented as mean ± SD, with *n* = 3. Statistical analysis was performed using ordinary one-way ANOVA followed by Tukey’s multiple comparison test. For each bacterial species, inhibition zones obtained at different C14R concentrations were compared to the highest tested concentration (100 µg/mL). Statistical significance relative to 100 µg/mL is indicated by asterisks (* *p* < 0.05, *** *p* < 0.001, **** *p* < 0.0001).

The antimicrobial activity of the synthetic peptide C14R against six clinically relevant ESKAPE pathogens was evaluated using the two-layer radial diffusion assay (Figure 1A,B). Clear inhibition zones were observed for all six strains, indicating that C14R exerts broad-spectrum antibacterial effects. The extent of inhibition varied among species, with *E. faecium* and *P. aeruginosa* showing larger inhibition zones, while *S. aureus* displayed the smallest one. To examine concentration-dependent responses to C14R in each bacterial species, inhibition zone diameters were compared by performing a one-way ANOVA and subsequent Tukey multiple comparison analysis. For clarity and biological relevance, all concentrations were compared to the highest peptide concentration (100 µg/mL), which served as the reference condition. This approach allowed the identification of concentrations at which antibacterial activity was significantly reduced relative to maximal inhibition.

**Figure 2:**
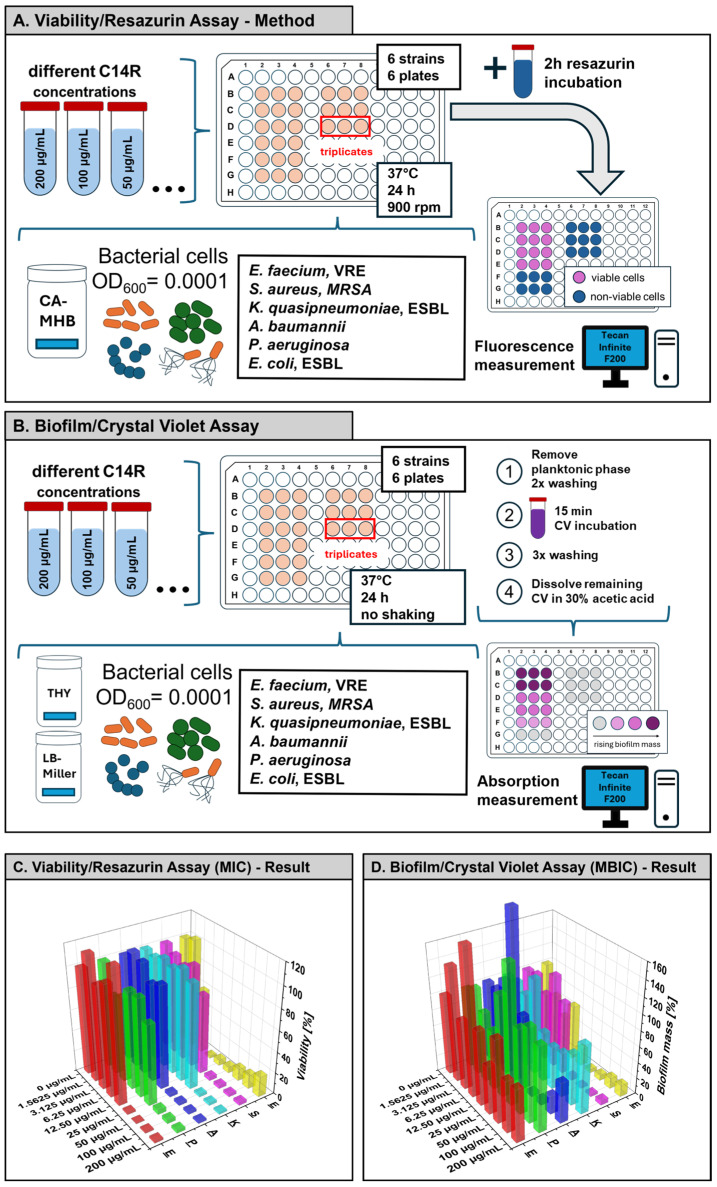
Experimental setup and results of antimicrobial and antibiofilm activity assays for C14R against ESKAPE pathogens. (**A**) Schematic overview of the resazurin-based viability assay with ESKAPE strains in the presence of C14R (0–200 µg/mL) using cation-adjusted Mueller–Hinton Broth (CAMHB) as the standard medium for susceptibility testing according to Clinical & Laboratory Standards Institute (CLSI) M100 guidelines [41]. (**B**) Schematic overview of the biofilm/crystal violet assay for measuring the biofilm mass in high nutrient medium, so either Todd–Hewitt (THY) medium or LB medium supplemented with C14R (0–200 µg/mL). Attached biofilms were stained with crystal violet and washed and solubilized for quantification. (**C**) Results of the resazurin-based susceptibility assay showing the viability of planktonic ESKAPE cells after 24 h exposure to increasing C14R concentrations (**D**) Results of the crystal violet biofilm assay showing relative biofilm formation during a 24 h exposition to different concentrations of C14R.

To further quantify the antimicrobial potency, resazurin-based viability assays were performed with increasing concentrations of C14R. A clear, concentration-dependent reduction in viability was detected for all pathogens (Figure 2A,C). The most susceptible strain was *E. faecium* (VRE) with a MIC of 3.44 µg/mL, followed by *S. aureus* (MRSA) with 14.72 µg/mL, *A. baumannii* (23.10 µg/mL) and *E. coli* (ESBL, 24.55 µg/mL). *P. aeruginosa* required slightly higher concentrations for complete inhibition (30.08 µg/mL), while *K. quasipneumoniae* (ESBL) showed the highest tolerance with an MIC of 45.15 µg/mL (Table 1). These results demonstrate that C14R shows wide-ranging yet variable activity against clinically important Gram-positive and -negative pathogenic strains. Since biofilm formation is a major virulence factor of ESKAPE pathogens [40], the effect of C14R on biofilm development was assessed using the crystal violet assay (Figure 2B). In contrast to the MIC assays, a nutrient-rich medium rather than a minimal medium was used for the MBIC assays to ensure optimal biofilm growth conditions, as biofilm formation in minimal medium was not sufficiently robust. This may account for the higher MBIC compared to the MIC values observed within the same strain. Biofilm biomass decreased in a dose-dependent manner for *E. faecium* and *S. aureus*, with a minimum biofilm inhibitory concentration (MBIC) for *E. faecium* of 22.05 µg/mL and *S. aureus* of 15.02 µg/mL (Table 1). For the other ESKAPE pathogens biofilm mass was not or slightly reduced but not consistently enough; therefore, no reliable MBIC values could be determined (not determinable, n.d., Table 1). Notably, *S. aureus* biofilms were strongly affected by C14R at intermediate concentrations, whereas *K. quasipneumoniae* was least responsive, reflecting its higher MIC value. These findings demonstrate that C14R possesses strong antibacterial activity against planktonic bacteria and partly against biofilm formation of ESKAPE pathogens, whereas the effect against the planktonic phase was higher against the Gram-positive (*E. faecium*, *S. aureus*) but also inhibited Gram-negative strains.

In summary, the quantitative analysis of the viability (MIC) and biofilm assays (MBIC) confirmed the activity of C14R against all ESKAPE pathogens, however, with different extents (Table 1). For several ESKAPE pathogens, including *K. quasipneumoniae*, *A. baumannii*, *P. aeruginosa* and *E. coli*, a concentration-dependent reduction in biofilm biomass was observed; however, complete inhibition of biofilm formation was not achieved within the tested C14R concentration range. Consequently, no MBIC could be determined for these species, and values are reported as not applicable (n.a.). This reflects the high intrinsic tolerance of Gram-negative ESKAPE biofilms rather than experimental failure.

**Figure 3:**
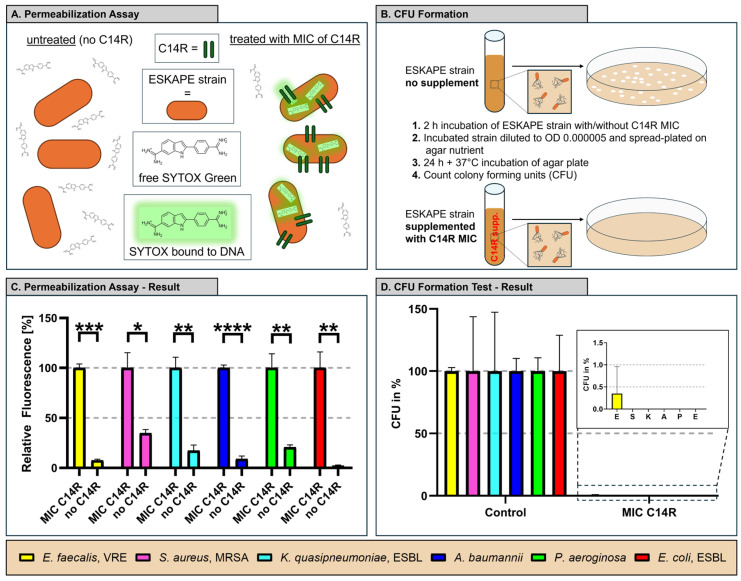
C14R MIC exposure strongly induces membrane permeabilization and reduces bacterial viability (CFU) in all ESKAPE pathogens. (**A**) Schematic representation of the SYTOX Green membrane permeabilization assay. ESKAPE strains were incubated with C14R, followed by addition of SYTOX Green dye, which selectively enters membrane-compromised cells and fluoresces upon binding to DNA. (**B**) Colony forming unit (CFU) assay setup. ESKAPE cultures were exposed for 25 h either to medium alone (control) or the C14R peptide at MIC concentrations, followed by plating the incubated cell on agar nutrient plates. After a 24 h incubation of the plates, CFUs were counted on control compared to C14R pretreated cells. (**C**) Membrane permeabilization of ESKAPE pathogens treated with the determined strain-specific C14R MIC. SYTOX Green uptake was measured afterwards, given that increased fluorescence indicates compromised membrane integrity. (**D**) Results of CFU formation testing. Data were analyzed with a standard one-way analysis of variance (ANOVA), followed by Tukey’s post hoc multiple comparison test. Significance levels were defined as * *p* < 0.05, ** *p* < 0.01, *** *p* < 0.001, **** *p* < 0.0001. Error bars indicate the mean ± standard deviation, reflecting variability among independent biological replicates.

The membrane permeabilization assay demonstrated that treatment of all ESKAPE pathogens with their respective strain-specific MICs of C14R resulted in pore formation, as indicated by a strong increase in SYTOX Green fluorescence signals across all tested species (Figure 3A,C). Since SYTOX Green selectively penetrates cells with compromised membranes and fluoresces upon DNA binding, these data indicate a loss of membrane integrity following C14R exposure. Correspondingly, colony forming unit (CFU) enumeration revealed a marked reduction in bacterial viability, with colony numbers being strongly decreased or nearly abolished compared to untreated controls (Figure 3B,D). The comparatively large standard deviations observed for certain pathogens reflect biological variability in CFU recovery following C14R exposure rather than technical inconsistencies. CFU enumeration was performed using standardized conditions and biological triplicates, and variability likely arises from heterogenous survival and recovery of subpopulations after membrane disruption, a phenomenon commonly observed for antimicrobial peptide treatments. Collectively, these results confirm that the determined MICs of C14R are biologically active and exert bactericidal effects against all ESKAPE pathogens.

## 3. Discussion

Antimicrobial peptides have gained increasing attention as alternative or complementary strategies to conventional antibiotics, particularly in the context of multidrug-resistant ESKAPE pathogens, which continue to represent a major burden in hospital-acquired infections worldwide [42,43,44]. The AMP C14R displayed robust activity against all six ESKAPE pathogens across different tests, confirming a broad antibacterial spectrum against these clinically highly relevant bacterial species. Importantly, the strains investigated in this study represent multidrug-resistant and antibiotic-priority pathogens that are frequently associated with difficult-to-treat nosocomial infections, thereby increasing the translational relevance of the presented data [2,4,45]. The use of harmonized and directly comparable assay conditions across all species enables a systematic evaluation of the activity spectrum of C14R that extends beyond previous pathogen-specific analyses. The findings are consistent with earlier C14R studies that classified the peptide as pore-forming and membrane-targeting, with potent activity against *P. aeruginosa* and low cytotoxicity towards lung fibroblasts [36]. Beyond antibacterial activity, C14R has been shown to act against fungal pathogens, including *Candida albicans*, *Candidozyma auris* (formerly *Candida auris*) and *Candida parapsilosis* [37,38]. Likewise, C14R exhibits antifungal activity against additional clinically relevant strains that are declared as high-priority species by the WHO. Their list contains strains like *Cryptococcus neoformans*, *Cryptococcus gattii*, *Nakaseomyces glabratus*, *Candida tropicalis*, *Pichia kudriavseveii* and *Candida dubliniensis*, further underscoring its broad-spectrum antimicrobial potential [39]. While these previous studies focused on selected individual pathogens, the present work extends the current knowledge by providing a systematic, side-by-side evaluation of C14R against the complete ESKAPE pathogens group under standardized experimental conditions. By directly comparing planktonic susceptibility, antibiofilm activity, membrane permeabilization and bactericidal effects across all six ESKAPE species, including antibiotic-resistant strains, this study established a unified susceptibility profile that highlights both the strengths and limitations of C14R in a clinically relevant context. Notably, the data reveals a higher susceptibility of Gram-positive ESKAPE pathogens compared to Gram-negative species, a finding that may guide future optimization and application strategies. The observed cross-kingdom activity of C14R, spanning major ESKAPE bacteria and clinically important fungi like all *Candida* species, suggest potential application niches where mixed bacterial–fungal infections are common (e.g., device-associated infections, chronic wounds, and blood stream infections) [46,47]. Benchmarking against another clinically advanced antimicrobial peptide pexiganan confirms that the MIC values obtained for C14R are within a relevant and literature-consistent range. In Flamm et al., pexiganan inhibited ESKAPE pathogens predominantly at 8–32 µg/mL, with low MICs for *E. faecium* (4–8 µg/mL) and *S. aureus* (MRSA/MSSA: 16–32 µg/mL), moderate MICs for *P. aeruginosa* and *A. baumannii* (8–16 µg/mL) and pronounced isolate-dependent variability among *Enterobacteriaceae*, including *K. pneumoniae* (MIC_90_ up to 128 µg/mL) [48]. In direct comparison, C14R exhibited comparable potency against Gram-positive ESKAPE members, with an MIC of 3.44 µg/mL for VRE *E. faecium* and 14.72 µg/mL for MRSA, closely matching reported pexiganan values. Against Gram-negative pathogens, C14R showed higher but still overlapping MICs. The elevated MIC observed for ESBL *K. quasipneumoniae* (45.15 µg/mL) is consistent with the known heterogeneity and high-MIC outliers reported for *Klebsiella* even for clinically developed AMPs such as pexiganan. Beyond the influence of nutrient-rich media, the pore-forming mechanism of C14R itself may contribute to the observed differences between planktonic cells and cells undergoing biofilm formation. In planktonic bacteria, C14R can readily access the cytoplasmic membrane, enabling rapid peptide insertion and pore formation that result in efficient membrane permeabilization and cell death [49]. In contrast, during the transition from planktonic growth to a biofilm-associated state, bacterial cells begin to produce extracellular polymeric substances and adopt surface-attached lifestyles, which can impede peptide diffusion, promote sequestration of cationic antimicrobial peptides and reduce the effective local concentration of C14R at the bacterial cell surface. But also with the biofilm burden, C14R could be used with combination strategies (with matrix-disrupting enzymes, chelators or antibiotics) and delivery formats (hydrogels) that increase local concentration and residence time [50,51]. Such formats have been investigated as an antimicrobial drug layer within a two-layer hydrogel, where the upper layer serves as a trapping zone, for example through the incorporation of high-affinity aptamer binding entities against *P. aeruginosa* [52]. While CFU enumeration revealed a pronounced reduction in bacterial viability across all species, the magnitude of SYTOX Green fluorescence signals varied between pathogens. Such species-dependent differences in fluorescence intensity are likely attributable to variations in membrane composition and surface architecture. Differences in membrane charge density, lipid composition and lipopolysaccharide structure can influence peptide–membrane interaction as well as the extent of SYTOX Green uptake following membrane permeabilization. Consequently, SYTOX Green fluorescence should be interpreted as a qualitative indicator of membrane disruption rather than a strictly quantitative surrogate for cell death when comparing phylogenetically distinct bacterial species. In this context of rising antimicrobial resistance among high-risk and clinically prioritized pathogens, the systematic dataset presented here positions C14R as a strong candidate compound for advancing the development of future antimicrobial approaches aimed at complementing or extending current therapeutic options.

## 4. Materials and Methods

### 4.1. Bacterial Strains and Cultivation

Liquid cultures of *E. coli*, *A. baumannii* and *P. aeruginosa* were grown in lysogeny-broth (LB–Miller) (Carl Roth GmbH + Co. KG, Karlsruhe, Germany) overnight at 37 °C with shaking (160 rpm). Other strains were cultured in Todd–Hewitt-broth (Oxoid Deutschland GmbH, Wesel, Germany) supplemented with 0.5% yeast extract (Becton, Dickinson and Company, Franklin Lakes, USA). All strains were obtained from the Institute of Medical Microbiology and Hygiene, University Clinic of Ulm: *Enterococcus faecium* (DSM 17050), *Staphylococcus aureus* (ATCC 43300), *Klebsiella quasipneumoniae* (ATCC 700603), *Acinetobacter baumannii* (ATCC 19606), *Pseudomonas aeruginosa* PAO1 and *Escherichia coli* (BSU1286). The bacterial strains included in this study are listed in Table 1. *E. coli and A. baumannii* were cultured in LB–Miller medium, and the remaining species were incubated in THY broth. *K. quasipneumoniae* recently got reclassified from *K. pneumoniae* to *K. quasipneumoniae* by the American Type Culture Collection (ATCC).

### 4.2. Antimicrobial Peptide C14R

The antimicrobial peptide was produced using solid-phase peptide synthesis and subsequently purified by reversed-phase high-performance liquid chromatography at the ULMTeC Core Facility for Functional Peptidomics as a part of Ulm University. Peptide identity and purity (>95%) were confirmed by mass spectrometry. Freeze-dried C14R was reconstituted in sterile phosphate-buffered saline to prepare stock solutions, which were divided into aliquots and kept at −20 °C. Fresh working solutions were obtained by diluting the stocks immediately before use.

### 4.3. Radial Diffusion Assay

Target bacteria were inoculated into liquid agarose at a density of 2 × 10^7^ cells per plate. Cultures were inoculated with 1 mL of washed overnight cultures. Wells, put into the solidified plate, were filled with C14R at concentrations ranging from 3.125 to 100 µg/mL. After 3 h incubation at 37 °C, plates were overlaid with tryptic soy agar and incubated overnight at 37 °C in 5% CO_2_. Inhibition zones were then measured.

### 4.4. Viability/Resazurin Assay

The viability of the ESKAPE pathogens in the presence of C14R (0–200 µg/mL) was assessed according to the CLSI guidelines for antimicrobial susceptibility testing (M100) [41] using Mueller–Hinton broth (MHB) (Carl Roth GmbH + Co. KG, Karlsruhe, Germany). Bacterial cultures were adjusted to OD_600_ = 0.0001 and inoculated into 200 µL of MHB supplemented with C14R in flat-bottomed 96-well plates. After this the well plates were incubated at 37 °C for 24 h and shaken at 900 rpm. Viability was determined using a modified procedure apart from the CSLI guidelines using a resazurin reduction assay, in which 20 µL of a 0.15 mg/mL resazurin solution was added and incubated for 2 h at 37 °C. Conversion of resazurin to resorufin was quantified fluorometrically (at wavelengths of λ_Ex_ = 535 nm and λ_Em_ = 595 nm) using a microplate reader of the model Tecan Infinite F200 from the company Tecan Group Ltd. in Männedorf, Switzerland [53]. Minimum inhibitory concentrations (MICs) of C14R were determined via Gompertz fit analysis (data provided in repository). All experiments were done in triplicate.

### 4.5. Biofilm/Crystal Violet Assay

Biofilm formation of ESKAPE pathogens was analyzed by performing a crystal violet assay originally established for bacterial biofilms by George O’Toole [54]. To start, an initial inoculum of OD_600_ = 0.0001 in Todd–Hewitt broth (*E. faecium*, *S. aureus*, and *K. quasipneumoniae*) and LB medium (*A. baumannii*, *E. coli*, and *P. aeruginosa*) was prepared. Biofilms were exposed to different concentrations of C14R (0–200 µg/mL) diluted in phosphate-buffered saline (PBS) (Life Technologies, Carlsbad, CA, USA). All experiments were done in triplicate.

### 4.6. Colony Forming Unit (CFU) Assay

Overnight cultures were adjusted to OD_600_ = 0.0001 and incubated for 2 h at 37 °C either without peptide (control) or with the strain-specific MIC concentration of C14R (Table 1) in 500 µL total volume. After 2 h incubation 100 µL of the incubated cells was plated on agar to prevent a bacterial lawn and incubated for 24 h at 37 °C. All experiments were done in triplicate.

### 4.7. SYTOX Green Membrane Permeabilization Assay

Membrane permeabilization was analyzed using the SYTOX Green assay. Overnight cultures were adjusted to OD_600_ = 0.1, centrifuged (4000× *g*, 5 min) and resuspended in 10 mM PBS containing 0.5 µM SYTOX Green (Thermo Fisher Scientific, Inc., Waltham, MA, USA). An amount of 90 µL of cells in the SYTOX Green solution was mixed with PBS only and C14R in PBS at the strain-specific MIC. After a 10 min incubation, fluorescence (excitation 488 nm/emission 530 nm) was measured using a Tecan Infinite M200 plate reader (Tecan Group Ltd., Männedorf, Switzerland). Longer C14R incubation did not show higher fluorescence values. All measurements were performed in triplicate.

### 4.8. Statistical Analysis

Each experiment was conducted with a minimum of three independent biological replicates. Data are presented as mean ± standard deviation (SD). Statistical analyses were conducted using GraphPad Prism 9. For the radial diffusion assay, inhibition zone diameters were analyzed separately for each bacterial species using ordinary one-way analysis of variance (ANOVA), followed by Tukey’s multiple comparison test. Comparisons were performed relative to the highest tested C14R concentration, which served as the reference condition. CFU enumeration data were analyzed using ordinary one-way ANOVA with a Tukey’s multiple comparison test afterwards to assess differences between untreated controls and C14R-treated samples. MICs and MBICs were not determined by statistical hypothesis testing but were calculated by nonlinear regression analysis using a Gompertz growth inhibition model fitted to concentration–response curves obtained from resazurin-based viability assays and crystal violet biofilm assays, respectively. MIC and MBIC values were defined as the peptide concentrations corresponding to complete growth or biofilm inhibition according to the fitted model. Due to the model-based nature of MIC and MBIC determination, no *p*-values were calculated for these parameters. Statistical significance was determined as follows: * *p* < 0.05, ** *p* < 0.01, *** *p* < 0.001, **** *p* < 0.0001.

## Figures and Tables

**Table 1:** Summary of MIC and MBIC of ESKAPE pathogens. MBIC values are reported as “n.a.” when complete inhibition of biofilm formation was not achieved at the highest tested C14R concentration. Partial biofilm reduction was observed for these species but did not meet the criteria required for MBIC determination. (n.a. = not applicable).

Microorganism	MIC [µg/mL]	MBIC [µg/mL]
*E. faecium*, VRE	3.44	22.05
*S. aureus*, MRSA	14.72	15.02
*K. quasipneumoniae*, ESBL	45.15	n.a.
*A. baumannii*	23.10	n.a.
*P. aeruginosa*	30.08	n.a.
*E. coli*, ESBL	24.55	n.a.

## Data Availability

Data available at https://data.mendeley.com/datasets/64vwvnpchv/1 (accessed on 9 February 2026).
